# Development of a risk prediction model (Hangang) and comparison with clinical severity scores in burn patients

**DOI:** 10.1371/journal.pone.0211075

**Published:** 2019-02-06

**Authors:** Youngmin Kim, Dohern Kym, Jun Hur, Jinwoo Jeon, Jaechul Yoon, Haejun Yim, Yong Suk Cho, Wook Chun

**Affiliations:** Department of Surgery and Critical Care, Burn Center, Hangang Sacred Heart Hospital, Hallym University Medical Center, Youngdeungpo-gu, Seoul, Korea; University of Mississippi Medical Center, UNITED STATES

## Abstract

**Purpose:**

The purpose of this study was to develop a new prediction model to reflect the risk of mortality and severity of disease and to evaluate the ability of the developed model to predict mortality among adult burn patients.

**Methods:**

This study included 2009 patients aged more than 18 years who were admitted to the intensive care unit (ICU) within 24 hours after a burn. We divided the patients into two groups; those admitted from January 2007 to December 2013 were included in the derivation group and those admitted from January 2014 to September 2017 were included in the validation group. Shrinkage methods with 10-folds cross-validation were performed to identify variables and limit overfitting of the model. The discrimination was analyzed using the area under the curve (AUC) of the receiver operating characteristic curve. The Brier score, integrated discrimination improvement (IDI), and net reclassification improvement (NRI) were also calculated. The calibration was analyzed using the Hosmer-Lemeshow goodness-of-fit test (HL test). The clinical usefulness was evaluated using a decision-curve analysis.

**Results:**

The Hangang model showed good calibration with the HL test (χ^2^ = 8.785, p = 0.361); the highest AUC and the lowest Brier score were 0.943 and 0.068, respectively. The NRI and IDI were 0.124 (p-value = 0.003) and 0.079 (p-value <0.001) when compared with FLAMES, respectively.

**Conclusions:**

This model reflects the current risk factors of mortality among adult burn patients. Furthermore, it was a highly discriminatory and well-calibrated model for the prediction of mortality in this cohort.

## Introduction

Prediction of critically ill patients in a systemic manner based on clear, objective data is an essential part of care in an Intensive Care Unit (ICU). The development of severity scoring systems has been transformed to predict outcomes in a more objective and reliable way and has sequentially influenced management decisions, including do-not-resuscitate status and the withdrawal of life support [1]. Severity scoring systems have continued to be developed and various scoring systems have been used for the critical ill patient. Severity scoring systems should have validity, calibration, and discrimination to predict the severity of disease and mortality, as well as repeatability and reliability in different populations and diseases [2, 3]. There are generally two kinds of prediction models due to the different characteristics of individual diseases. One is used for the general intensive care patients and is focused on the acute physiological status and associated comorbidities assessed by the Acute Physiologic and Chronic Health Evaluation (APACHE) II [4], Simplified Acute Physiologic Score (SAPS) II [5], Logistic Organ Dysfunction Score (LODS) [6], and Sequential Organ Failure Assessment (SOFA) [7]. The other is specific to each individual disease and consists of the disease-related features. Among burn patients, the abbreviated burn severity index (ABSI) [8], FLAMES (Fatality by Longevity, APACHE II score, Measured Extent of burn, and Sex) [9], the revised Baux index (rBaux) [10], the models which were developed by Ryan et al [11], and the Belgian outcome in burn injury (BOBI)[12] group are known and used widely. These burn-specific prediction models, with the exception of FLAMES, consist of patient-related factors; no laboratory variables are included and even in FLAMES there is not burn specific laboratory factors. Therefore, these models are only able to determine some of the risk factors for mortality rather than a continuous range of risk factors [9]. It is necessary to develop a prediction model that includes a wider range of treatment-related biological variables as well as patient-related variables to accurately reflect the rapid progress in burn treatment. Additionally, the existing scoring systems for the general critically ill patients do not accurately predict the severity and the risk of mortality in the burn patients because they were developed from the general ICU, and did not specifically take into consideration burn populations [13].

The purpose of this study was to develop a new prediction model for mortality among burn patients that included specific laboratory tests to better reflect the risk of mortality and severity of disease as survival rates have increased due to the development of burn treatment in recent years. Additionally, we aimed to evaluate whether the newly developed prediction model could predict the risk of mortality more accurately than existing scoring systems.

## Materials and methods

### Patients

This study included 2009 patients aged more than 18 years who were admitted within 24 hours after a burn in the burn intensive care unit (BICU) of Hangang Sacred Heart Hospital, Hallym University Medical Center from January 2007 to September 2017. The criteria for admission to BICU were as follows; 1) partial thickness burn of more than 20% of total body surface area (TBSA) for adults and partial thickness burn of more than 10% of TBSA if the patient was over 65 years of age, 2) inhalation injury, 3) electrical burn, 4) pre-existing medical disorder that could incur complications, or affect mortality, and 5) with concomitant trauma, which could elevate the risk of the morbidity or mortality. We divided the patients into two groups to develop and validate the new Hangang model; the patients who were admitted from January 2007 to December 2013 were included in the derivation group and the patients who were admitted form January 2014 to September 2017 were included in the validation group. This study was approved by the Institutional Review Board of Hangang Sacred Heart Hospital. Informed consent was waived due to the retrospective nature of the study.

### Variables and prediction models

All medical records of patients were retrieved from the clinical data warehouse which stored all electronic medical records anonymously in Hallym University Medical Center. The following demographic variables were collected; age, sex, type of burn, percentage of TBSA (% TBSA) burned, presence of inhalation, pre-existing medical history. There were no missing data; all subjects had complete data. The outcome of the prediction models was the 60-day mortality. We evaluated 10 prediction models. Five models such as ABSI [8], Ryan [11], FLAMES[9], BOBI [12] and the revised Baux [10] which are specific for burn patients and the most well-known [2], were calculated from electronic medical records. The ABSI scores consisted of five variables; age (1–5 points), % TBSA burned (1–10 points), female gender (1 point), the presence of inhalation injury (1 point), and the presence of full-thickness burn (1 point). The Ryan score was the sum of the presence of three risk factors (greater than 60 years of age, greater than 40% TBSA, and the presence of inhalation injuries). The FLAMES score was calculated using age, the percentage of partial and full thickness burns, gender, and the APACHE II score on day 1. The BOBI score was calculated using age (0–3 points), % TBSA burned (0–4 points), and the presence of inhalation injury (3 points). The revised Baux score was calculated using age + % TBSA burned + 17 (presence of inhalation injury). Inhalation injuries were diagnosed based on the patients’ history (burned in a closed space, unconscious at the scene, prolonged extrication), physical findings such as singed facial hair, carbonaceous deposits in the nose or mouth, or facial burns, and other diagnostic modalities such as bronchoscopy, carbon monoxide levels, and serial chest x-rays. The presence of medical comorbidities was identified based on the presence of one or more of the following; cardiac disease, liver or kidney disease, and diabetes mellitus. APACHE II[4], SPAS II[5], LODS[6], SOFA[7] which are models generally used for the critical ill patients, were also calculated using the electronic medical records. APACHE II and SAPS II, LODS score, and SOFA consisted of 12, 11, and six physiologic variables [3]. All laboratory variables were used in these prediction models; the models retrieved the worst value of laboratory variables during first 24 hours after admission.

### Burn management

All patients who were admitted to BICU received initial fluid resuscitation using the modified Parkland formula (4 mL × kg × % TBSA burned); the fluid volume was adjusted as needed to maintain a minimum urine output of 0.5 mL/kg/hour. Enteral feeding was the first choice and initiated within 48 hours if there was no ileus; parenteral nutrition was supplemented to meet the target caloric requirements, which were measured using the European society of parenteral and enteral nutrition (ESPEN) guidelines for intensive care [14]. Burn wound dressing was conducted daily using hydrofoam and topical antimicrobials. Early excision and grafting with auto-/allograft was performed within 5 days after admission.

### Statistical analysis

Continuous variables distributed normally and non-normally were presented as means ± standard deviation (SD) and as medians (25^th^ interquartile range [IQR] - 75^th^ IQR), respectively. The paired t-test or Wilcoxon signed rank test, depending on the normality of the data, was used to determine differences between the two groups. Categorical variables are presented as proportions and differences between them were analyzed using Chi-square tests. Two side p-values <0.05 were considered statistically significant. All statistical analyses were conducted using the computing statistical R-project program version 3.5.1.

### Development of the new prediction model (Hangang)

The following 30 variables were obtained, including eight patients’ variables (six demographic values, participants medical histories, and the Glasgow coma scale) and 22 physiologic values (Table 1). To detect multicollinearity for the all variables in this model, we used variance inflation factors. Shrinkage methods (the least absolute shrinkage and selection operator [LASSO]) with 10-folds cross-validation were performed using the computing statistical R-project program with the ‘glmnet’ package to determine the least number of variables for the development of the model and to limit overfitting of the model. Then, ten variables including age, % TBSA burned, inhalation injuries, serum lactate, pH, prothrombin time (PT), serum bilirubin, serum myoglobin, serum creatinine, and lactate dehydrogenase (LD) were included finally in the Hangang model.

**Table 1:** The logistic regression coefficients and the assigned points for categorized variables included in the new prediction model.

	Coefficient	p-value	Weight	Weight Scalded		Points
Intercept	-6.674	< 0.001	-48.146	0.000	0	
Age ≤ 50	0.000		0.000	10.024	10	
Age 51–65	1.389	< 0.001	10.017	20.041	20	
Age > 65	3.181	< 0.001	22.943	32.968	33	
% TBSA burned ≤ 20	0.000		0.000	10.024	10	
% TBSA burned 21–40	1.060	0.002	7.646	17.670	18	
% TBSA burned 41–65	2.136	< 0.001	15.407	25.431	25	
% TBSA burned 66–75	3.659	< 0.001	26.392	36.416	36	
% TBSA burned > 75	5.811	< 0.001	41.919	51.944	52	
No inhalation	0.000		0.000	10.024	10	
Inhalation	0.422	0.091	3.045	13.070	13	
Lactate ≤ 2.0	0.000		0.000	10.024	10	
Lactate 2.1–4.0	0.659	0.057	4.751	14.775	15	
Lactate > 4.0	1.322	<0.001	9.534	19.559	20	
pH ≤ 7.25	0.000		0.000	10.024	10	
pH 7.26–7.35	-0.712	0.041	-5.135	4.889	5	
pH > 7.35	-1.216	0.001	-8.772	1.253	1	
PT ≤ 11	0.000		0.000	10.024	10	
PT 12–14	0.762	0.005	5.500	15.524	16	
PT > 14	1.618	<0.001	11.675	21.699	22	
Bilirubin ≤ 1.2	0.000		0.000	10.024	10	
Bilirubin 1.3–2.4	0.400	0.114	2.885	12.909	13	
Bilirubin > 2.4	0.640	0.123	4.619	14.643	15	
Serum myoglobin ≤ 65	0.000		0.000	10.024	10	
Serum myoglobin 66–145	0.359	0.352	2.592	12.617	13	
Serum myoglobin > 145	0.912	0.005	6.575	16.600	17	
Creatinine ≤ 1.2	0.000		0.000	10.024	10	
Creatinine > 1.2	0.591	0.053	4.260	14.284	14	
LD ≤ 300	0.000		0.000	10.024	10	
LD 301–750	0.633	0.128	4.568	14.592	15	
LD > 750	1.405	0.003	10.138	20.162	20	

%TBSA burn, the percent of total body surface area burned; n, number; FB, flame burn; SB, scald burn; EB, electrical burn; ChB, chemical burn; PT, prothrombin time; LD, lactate dehydrogenase

The continuous features were divided into groups which were mapped to a target variable (mortality) by supervised discretization using algorithms such as Recursive Partitioning, which can identify optimal cut points and evaluate the relationship with the outcome using the Weight of Evidence and Information Values [15]. The optimal cut points were adjusted to ensure the model was simple and easy to interpret. Then the variables were categorized by the adjusted cut points. The points were assigned to the categorized variable using the coefficients calculated using the computing statistical R-project program with the ‘smbinning’ package (Table 1). A nomogram of the Hangang model shows the scores of each variable (Table 2). The minimum and maximum scores of the Hangang model ranged from 91 to 216 and the probability (%) of mortality according to scores (Table 3).

**Table 2:** The score for each variable and probability of mortality in the new prediction model (Hangang).

Score	1	5	10	13	14	15	16	17	18	20	22	25	33	36	52
Age(years)			≤ 50							≤65			>65		
%TBSA burned			≤ 20						≤40			≤65		≤75	>75
Inhalation injury			No	Yes											
lactate(mmol/L)			≤ 2.0			≤4.0				>4.	0				
pH	>7.35	≤ 7.35	≤ 7.25												
PT(sec)			≤11.0				≤14.0				>14.0				
bilirubin(mg/dL)			≤1.2	≤2.4		>2.4									
Myoglobin(ng/mL)			≤65	≤145				>145							
Creatinine(mg/dL)			≤1.2		>1.2										
LD(IU/L)			≤300			≤750				>750					

%TBSA burned, the percentage of total body surface area burned; PT, prothrombin time; LD, lactate dehydrogenase

**Table 3:** Predicted mortality according to scores.

Total Scores	91	128	133	141	149	157	165	216
Mortality risk(%)	0%	5%	10%	25%	50%	75%	90%	100%

### Model performance

The discrimination was analyzed by the area under the curve (AUC) of the receiver operating characteristic curve (ROC); as the AUC approaches one, the discriminating power increased [16]. The Brier score was calculated; a Brier score of 0 indicates total accuracy [17]. The integrated discrimination improvement (IDI) and the net reclassification improvement (NRI) were also calculated using category options between the Hangang and other existing models using the computing statistical R-project program with the ‘PredictABEL’ package. The calibration was analyzed using the Hosmer-Lemeshow goodness-of-fit test (HL test), which assesses how well the mortality pattern in the data under analysis is described; non-significant p-values indicated that the fit of the model was good [18]. Clinical usefulness, or the ability to make better decisions with a model than without, was not assessed by discrimination and calibration [19]. Therefore, we also performed a decision-curve analysis. The code and manual for the decision-curve analysis is publicly available (www.decisioncurveanalysis.org).

## Results

### Comparison of baseline characteristic between the derivation group and validation group

In total, 2009 patients were included in this study; they were then divided into the derivation (n = 1406) and validation (n = 603) groups. The overall median age was 47.0 (38.0–56.0) years and participants were older in the validation group than in the derivation group (49.0 years vs 46.0 years, p = 0.003). The overall % TBSA burned was 30.0%; there was no significant difference between the two groups based on % TBSA burned (p = 0.127). Inhalation injuries were significantly more frequent in the derivation group (57.3% vs 51.1%, p = 0.011) and the patients in the validation group had more medical comorbidities (46.8% vs 21.0%, p<0.001). Overall mortality was 21.7% and there was no significant difference between the two groups. The validation group had significant differences in patient characteristics with the exception of sex (p = 0.409) and the type of burns (p = 0.099). The scores of the prediction models such as ABSI, rBaux, Ryan BOBI, FLAMES for burn patient did not significantly differ between the two groups. Only the SOFA scores for the prediction models for ICU patients did not significantly differ (Table 4). All physiologic variables are shown in the Table 4.

**Table 4:** Comparison of the baseline characteristics between the derivation and validation groups.

	Total (n = 2009)	Derivation (n = 1406)	Validation (n = 603)	p-value
Patient characteristics				
Age (years)	47.0(38.0–56.0)	46.0(38.0–55.0)	49.0(38.0–58.0)	0.003
Sex (male, %)	1656(82.4%)	1152(81.9%)	504(83.6%)	0.409
Type(FB:SB:EB:ChB:CoB)	1493:161:276:30:49	1061:112:180:17:36	432:49:96:13:13	0.099
%TBSA burned	30.0(17.0–50.0)	30.0(18.0–50.0)	29.0(16.0–46.0)	0.127
Inhalation Injury	1114(55.5%)	806(57.3%)	308(51.1%)	0.011
Medical History	577(28.7%)	295(21.0%)	282(46.8%)	<0.001
GCS	15.0(15.0–15.0)	15.0(15.0–15.0)	15.0(11.0–15.0)	<0.001
Physiologic variables				
MAP(mmHg)	86.0(73.0–99.0)	86.0(73.3–99.0)	86.3(72.5–98.0)	0.777
Heart rate(	82.0(70.0–98.0)	83.0(70.0–99.0)	81.0(70.0–98.0)	0.458
Respiratory rate	21.0(20.0–24.0)	22.0(20.0–24.0)	21.0(19.0–24.0)	0.002
Temperature(°C)	37.0(36.5–37.6)	37.0(36.5–37.5)	37.0(36.5–37.6)	0.233
PF ratio	275.7(204.0–348.3)	269.8(202.0–336.7)	290.0(212.9–398.8)	<0.001
Sodium(mEq/L)	141.0(138.0–143.0)	141.0(139.0–144.0)	140.0(138.0–142.0)	<0.001
Potassium(mEq/L)	4.2(3.9–4.6)	4.2(3.9–4.7)	4.1(3.8–4.6)	0.020
Urine output(ml/day)	1000.0(530.0–1665.0)	1000.0(530.0–1660.0)	970.0(500.0–1685.0)	0.699
BUN(mg/dL)	15.0(11.8–18.8)	15.2(12.0–19.0)	14.5(11.4–18.1)	0.003
Creatinine(mg/dL)	0.8(0.7–1.0)	0.8(0.7–1.0)	0.8(0.7–1.0)	0.715
pH	7.4(7.3–7.4)	7.4(7.3–7.4)	7.4(7.3–7.4)	<0.001
Bicarbonate(mmol/L)	20.9(18.3–22.8)	21.1(18.6–22.7)	20.1(17.6–23.1)	0.096
WBC(10^3^/uL)	15.3(11.2–20.9)	15.6(11.6–21.7)	14.6(10.4–19.3)	<0.001
Hct(%)	47.6(43.2–53.3)	47.6(43.2–53.4)	47.7(43.4–53.1)	0.844
Platelet(10^3^/uL)	222.0(172.0–279.0)	228.0(179.0–284.0)	209.0(163.0–265.5)	<0.001
Bilirubin(mg/dL)	0.8(0.6–1.2)	0.8(0.6–1.3)	0.8(0.5–1.1)	<0.001
PT(sec)	11.8(11.0–12.9)	11.4(10.8–12.3)	12.7(11.8–13.6)	<0.001
Lactate(mmol/L)	2.9(1.8–4.9)	2.8(1.7–4.7)	3.3(2.2–5.4)	<0.001
CK(IU/L)	300.0(158.0–1103.0)	315.5(164.0–1093.0)	278.0(148.5–1117.5)	0.147
LD(IU/L)	410.0(287.0–620.0)	440.5(307.0–656.0)	341.0(251.0–530.5)	<0.001
Serum myoglobin(ng/mL)	161.0(53.0–799.0)	176.5(60.0–911.0)	114.0(38.0–680.0)	<0.001
Urine myoglobin(+)	695(34.6%)	399(28.4%)	296(49.1%)	<0.001
Severity systems for burn				
ABSI	8.0(6.0–10.0)	8.0(6.0–10.0)	8.0(6.0–10.0)	0.071
rBaux	88.0(69.0–111.0)	88.0(69.0–112.0)	87.0(68.0–109.5)	0.434
Ryan	1.0(0.0–2.0)	1.0(0.0–2.0)	1.0(0.0–2.0)	0.068
BOBI	4.0(2.0–5.0)	4.0(2.0–5.0)	3.0(1.0–5.0)	0.235
FLAMES	-3.1(-4.8–0.6)	-3.2(-4.9–0.7)	-2.9(-4.8–0.4)	0.224
Hangang	126.0(114.0–143.0)	125.0(113.0–143.0)	127.0(116.0–145.0)	0.018
Severity system in ICU				
APACHE II	9.0(6.0–14.0)	8.0(6.0–13.0)	10.0(6.0–17.0)	<0.001
SAPS2	17.0(10.0–27.0)	16.0(9.0–25.0)	20.0(11.0–32.0)	<0.001
LODS	2.0(0.0–5.0)	1.0(0.0–5.0)	3.0(0.0–5.0)	<0.001
SOFA	2.0(2.0–4.0)	2.0(2.0–4.0)	3.0(2.0–5.0)	0.366
Mortality	435(21.7%)	305(21.7%)	130(21.6%)	0.994

n, number; FB, Flame Burn; SB, Scald Burn; EB, Electrical Burn; ChB, Chemical Burn; CoB, Contact Burn; %TBSA burned, percentage of total body surface area burned; MAP, Mean Arterial Pressure; PF ratio, ratio of arterial O_2_ pressure to fraction of inspired oxygen; WBC, White Blood Cell; Hct, Hematocrit; PT, Prothrombin Time; CK, Creatine Kinase; LD, Lactate Dehydrogenase; GCS, Glasgow Coma Scale; ABSI, Abbreviate Burn Severity Index; rBaux, revised Baux index; BOBI, Belgian Outcome in Burn Injury; FLAMES, Fatality by Longevity, APACHE II score, Measured Extent of burn, and Sex; APACHE, Acute Physiology and Chronic Health Evaluation Score; SAPS, Simplified Acute Physiology Score; LODS, Logistic Organ Dysfunction Score; SOFA, Sequential Organ Failure Assessment

### Validation of the existing models in the derivation cohort

Prior to validation of the new model, we evaluated the performance measures of nine existing scoring systems to determine their predictive ability. The AUC was the highest (0.957) and the Brier score was the lowest (0.071) in the Hangang model when compared with other models. The NRI and IDI were 0.504 (p-values <0.001) and 0.045 (p-values <0.001) for Hangang when compared with FLAMES. Hangang (HL test, χ^2^ = 8.354, p = 0.400), FLAMES (HL test, χ^2^ = 4.973, p = 0.760), and the Ryan score (HL test, χ^2^ = 0.359, p = 0.549) had significantly better calibration among all the models tested for burn patients and SAPS II (HL test, χ^2^ = 8.958, p = 0.346) had significantly better calibration among the models tested for ICU patients (Table 5).

**Table 5:** Performance measures of existing prediction model in derivation group.

Score	AUC (95% CI)	p-value	NRI (95%CI)	p-value	IDI (95% CI)	p-value	Brier score	Hosmer-Lemeshow χ^2^	p-value
Hangang	0.958 (0.947–0.968)	Reference	Reference	Reference	Reference	Reference	0.063	5.271	0.728
For burns									
FLAMES	0.939 (0.924–0.954)	0.001	0.036 (-0.008–0.081)	0.108	0.048 (0.024–0.072)	<0.001	0.071	7.434	0.491
rBaux	0.921 (0.903–0.939)	<0.001	0.071 (0.024–0.118)	0.003	0.109 (0.084–0.134)	<0.001	0.081	18.092	0.021
ABSI	0.906 (0.886–0.926)	<0.001	0.096 (0.046–0.146)	<0.001	0.135 (0.108–0.162)	<0.001	0.085	17.010	0.017
BOBI	0.885 (0.864–0.906)	<0.001	0.156 (0.108–0.203)	<0.001	0.216 (0.189–0.243)	<0.001	0.097	71.937	<0.001
Ryan	0.844 (0.824–0.865)	<0.001	0.555 (0.498–0.612)	<0.001	0.314 (0.282–0.347)	<0.001	0.117	0.359	0.549
In ICU									
APACHE II	0.884 (0.864–0.904)	<0.001	0.246 (0.178–0.314)	<0.001	0.262 (0.224–0.301)	<0.001	0.109	24.704	0.002
SAPS2	0.857 (0.834–0.880)	<0.001	0.275 (0.200–0.349)	<0.001	0.301 (0.257–0.344)	<0.001	0.114	8.958	0.346
SOFA	0.834 (0.806–0.861)	<0.001	0.321 (0.254–0.389)	<0.001	0.321 (0.280–0.362)	<0.001	0.117	15.747	0.003
LODS	0.786 (0.756–0.815)	<0.001	0.381 (0.309–0.453)	<0.001	0.412 (0.370–0.454)	<0.001	0.132	28.765	<0.001

AUC, Area Under the Curve; FLAMES, Fatality by Longevity, APACHE II score, Measured Extent of burn, and Sex; rBaux, revised Baux index; ABSI, Abbreviate Burn Severity Index, BOBI, Belgian Outcome in Burn Injury; ICU, Intensive Care Unit; APACHE, Acute Physiology and Chronic Health Evaluation Score; SAPS, Simplified Acute Physiology Score; SOFA, Sequential Organ Failure Assessment; SOFA, Sequential Organ Failure Assessment; LODS, Logistic Organ Dysfunction Score

### Validation of the new model (Hangang) in the validation cohort

**Fig 1:**
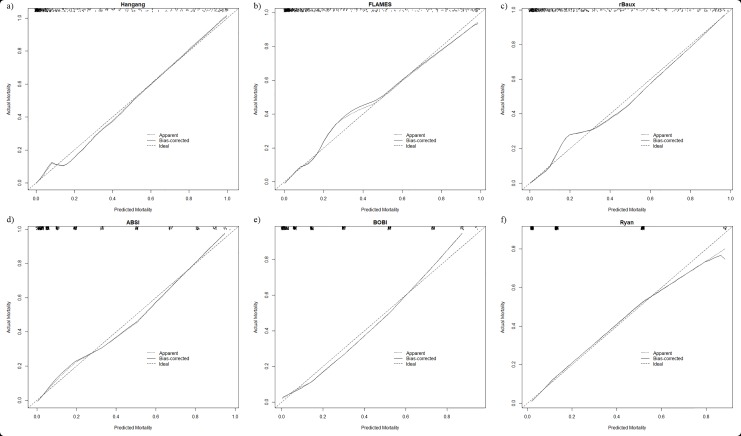
Calibration plots for the Hangang and models specific for burn patients in the validation group. Hosmer-Lemeshow goodness of fit statistics are presented in Table 6.

**Fig 2:**
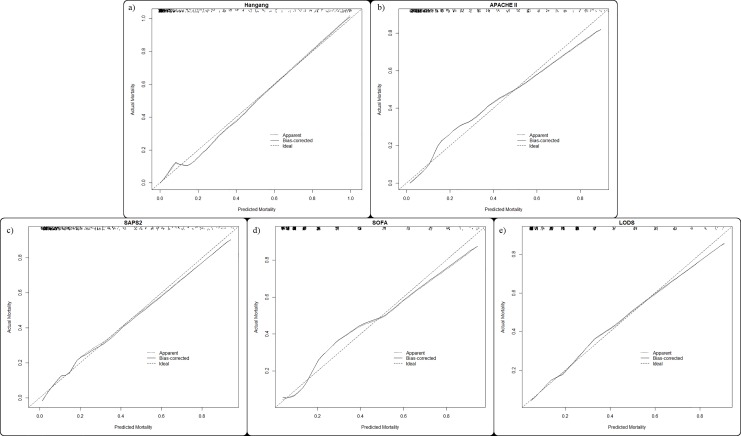
Calibration plots for the Hangang and models for patients in the intensive care unit. Hosmer-Lemeshow goodness of fit statistics are presented in Table 6.

**Fig 3:**
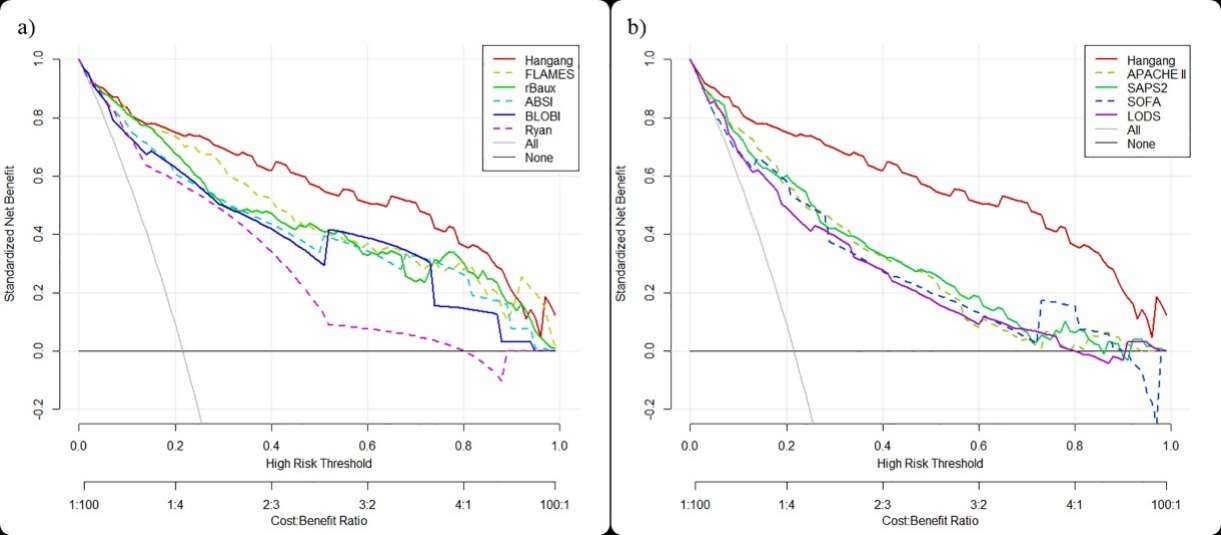
Decision curve analysis for the Hangang model compared with 1) models specific for burn patients, 2) models for patients in the intensive care unit.

Even though the demographic and physiologic characteristics of the validation group differed compared to the derivation group, the Hangang model showed improved prediction of the risk of mortality. Hangang had better calibration (HL test, χ^2^ = 8.785, p = 0.361) for predicting mortality; this was reinforced by the highest AUC (0.943) and the lowest Brier score (0.068). The NRI and IDI when compared with FLAMES were 0.124 (p = 0.003) and 0.079 (p-values <0.001), respectively. Among the prediction models tested for ICU patients, SAPS II had the highest AUC (0.860), an accuracy of 0.786, the lowest Brier score (0.115), and a HL test χ^2^ of 6.489 (p = 0.593) (Table 6). The calibration plots for all the existing models included in this study are shown in the Figs 1 and 2. The decision-curve indicates that the Hangang model was the best for predicting the probability of mortality (Fig 3).

**Table 6:** Performance measures of new Hangang model comparing with existing prediction model in validation group.

Score	AUC (95% CI)	p-value*	NRI (95% CI)	p-value	IDI (95% CI)	p-value	Brier score	Hosmer-Lemeshow χ^2^	p-value
Hangang	0.943 (0.921–0.966)	Reference	Reference	Reference	Reference	Reference	0.068	8.785	0.361
For burns									
FLAMES	0.927 (0.904–0.951)	0.090	0.124 (0.042–0.206)	0.003	0.079 (0.038–0.120)	<0.001	0.084	11.991	0.152
rBaux	0.914 (0.889–0.940)	0.008	0.118 (0.035–0.202)	0.005	0.122 (0.079–0.165)	<0.001	0.091	9.391	0.310
ABSI	0.893 (0.864–0.923)	<0.001	0.123 (0.29–0.217)	0.011	0.160 (0.114–0.206)	<0.001	0.097	6.640	0.355
BOBI	0.887 (0.856–0.917)	<0.001	0.126 (0.034–0.217)	0.007	0.185 (0.139–0.231)	<0.001	0.099	18.262	0.006
Ryan	0.841 (0.809–0.874)	<0.001	0.103 (0.011–0.195)	0.028	0.287 (0.234–0.340)	<0.001	0.118	1.373	0.241
In ICU									
APACHE II	0.859 (0.825–0.894)	<0.001	0.241 (0.135–0.347)	<0.001	0.273 (0.215–0.331)	<0.001	0.117	11.308	0.185
SAPS2	0.860 (0.826–0.893)	<0.001	0.253 (0.147–0.360)	<0.001	0.267 (0.204–0.330)	<0.001	0.115	6.489	0.593
SOFA	0.827 (0.786–0.869)	<0.001	0.317 (0.208–0.426)	<0.001	0.307 (0.244–0.370)	<0.001	0.122	3.230	0.665
LODS	0.823 (0.784–0.862)	<0.001	0.329 (0.222–0.436)	<0.001	0.335 (0.271–0.400)	<0.001	0.126	10.802	0.055

*, p-value compared with new Hangang model

AUC, Area Under the Curve; FLAMES, Fatality by Longevity, APACHE II score, Measured Extent of burn, and Sex; rBaux, revised Baux index; ABSI, Abbreviate Burn Severity Index, BOBI, Belgian Outcome in Burn Injury; ICU, Intensive Care Unit; APACHE, Acute Physiology and Chronic Health Evaluation Score; SAPS, Simplified Acute Physiology Score; SOFA, Sequential Organ Failure Assessment; SOFA, Sequential Organ Failure Assessment; LODS, Logistic Organ Dysfunction Score

## Discussions

Despite the existence of several prediction models, there are not many realistic models to accurately predict the outcomes of burn patients [20]. Various prediction models suggest that there is no ideal model to predict outcomes accurately in every population [2]. The ideal prediction model generally is simple, reliable, and objective (observer independent) [13]. However, in most burn-specific prediction models, it might be difficult to accurately reflect the risk of mortality, which has been changed as a result of the advancement of burn treatment. This is due to the fact that these models consist of patient-related variables (such as age and % TBSA) and do not contain objective laboratory values [21]. The % TBSA burned was the most powerful predictor in this study, however, it is measured differently based on the experience of the treating physician; the estimation error can be up to 20% among inexperienced physicians [22]. Therefore, in hospitals that are not specialized in treating burn patients, such errors can affect the model and make it difficult to accurately predict mortality. To compensate for these errors, prediction models should include the addition of objective laboratory results.

We assessed the validation of prediction models by calibration and decimation. Additionally, we assessed the ability to make better decisions with a model than without by conducting a decision-curve analysis [23]. The Hangang model showed that the net benefit (NB) was higher than other prediction models for patients in ICU and higher, with the exception of extremes, than other models for burn patients. These findings suggest that the Hangang model assists in making better decisions for the prediction of mortality.

Our model ensured accuracy, reliability, and objectivity by adding seven variables (lactate, pH, creatinine, PT, bilirubin, LD, serum myoglobin) associated with treatment over three variables (age, % TBSA burned, inhalation injury) which are commonly applied to existing burn specific prediction models, with the exception of FLAMES. Our model showed superiority when compared to the other existing models.

Among the laboratory variables included in this model, serum myoglobin and LD were not used as predictors for mortality in other prediction models. Serum myoglobin is associated with the burn depth and severity of the burn; previous studies have shown that burn patients with high myoglobinemia have a high risk of mortality [24, 25]. LD is also associated with burn diseases and mortality in patients with major burns [25–27]. When compared to FLAMES, which includes other physiologic variables (a form of APACHE II) similar to our Hangang model, we inferred that the Hangang model would have better prediction ability because burn-specific serum myoglobin and LD were included and other non-significant variables were excluded. Prediction models for the general ICU such as APACHE II, SAPS II, SOFA, and LODS showed poor predictability in critical burn patients. Therefore, caution should be taken when applying general prediction models to burn patients because they do not take into account the profound physiological effects of the burn itself, although they may prove valid in a general critical ill patients.[20]

This study was subject to several limitations. First, we did not validate the Hangang prediction score externally at other hospitals, because our burn center is the only burn center run by the Hallym university and has been designated as “The Emergency Center for Burn Care” by the Ministry for Health, Welfare, and Family Affairs in South Korea. However, validation was performed in the cohort recently treated in our center. Second, our study group did not include pediatric burn patients due to their different physiologic characteristics. Further studies including pediatric burn patients are needed. Third, not all patients who were admitted to BICU were included in this study; only acute burn patients who were admitted within 24 hours after injury were included in order to exclude other confounding factors. Fourth, although we collected the worst laboratory value over 24 hours for laboratory variables to minimize other affecting factors, the seven variables included in our model might have been affected by the level of fluid resuscitation, thus affecting our model.

Despite these limitations, it is important to recognize that our model reflects outcomes as a result of care provided under the current standards. Although our Hangang prediction model was developed in a single center, this is the largest study, to our knowledge, to date to test a new model for the prediction of mortality among burn patients. In the future, it might require modification to assist with decision-making as new therapies are introduced. We advocate that physicians who do not have much experience treating burns should consult experienced doctors when using this prediction model.

## Conclusions

There are many severity scoring systems widely used in the ICU to predict outcomes and characterize the severity of the disease. All of these scoring systems have been developed for the mixed population in the ICU. Their accuracy among subgroups, such as burn patients, is questionable and therefore, burn-specific scoring systems are required for accurate prediction. This model reflects the burn specific risk factors such as serum myoglobin and LD as well as current risk factors for mortality; it is a highly discriminatory and well-calibrated model for the prediction of mortality in adult burn patients.

## Supporting information

S1 Dataset Datasets of this study. (CSV)
